# Assessment of the Mechanical Properties of Different Suture Materials for Oral Surgery: An In Vitro Tensile Strength Study

**DOI:** 10.7759/cureus.65952

**Published:** 2024-08-01

**Authors:** Rajbir Kaur Randhawa, Tushar Dubey, Ishita Pansuriya, Tanisha Mishra, Monika Tanwar, Ankit Kumar, Ramanpal Singh

**Affiliations:** 1 Department of Oral and Maxillofacial Surgery, Government Dental College and Hospital, Jamnagar, IND; 2 Department of Oral and Maxillofacial Surgery, ITS Dental College and Research Centre, Ghaziabad, IND; 3 Department of Dentistry, Kalinga Institute of Dental Sciences, Kalinga Institute of Industrial Technology (KIIT) Deemed to be University, Bhubaneswar, IND; 4 Department of Oral and Maxillofacial Surgery, Faculty of Dental Sciences, Shree Guru Gobind Singh Tricentenary (SGT) University, Gurugram, IND; 5 Department of Oral and Maxillofacial Surgery, Kothiwal Dental College and Research Centre, Moradabad, IND; 6 Department of Oral Medicine and Radiology, New Horizon Dental College and Research Institute, Chhattisgarh, IND

**Keywords:** wound closure, in vitro study, tensile strength, suture materials, oral surgery

## Abstract

Background: Sutures are essential components of wound closure in oral surgery, and the mechanical properties of suture materials play a crucial role in determining surgical outcomes. Understanding the tensile strengths of various suture materials is vital for selecting the most appropriate material for specific clinical applications.

Objective: This study aimed to assess the tensile strength of suture materials commonly used in oral surgery through an in vitro tensile strength study.

Methods: A total of 192 samples of six commonly used suture materials (polyglycolic acid (PGA), polyglactin 910 (PGLA), polylactic acid (PLA), polydioxanone (PDO), silk, and nylon) were subjected to tensile strength testing using a universal testing machine. Descriptive statistics were used to summarize the tensile strength of each suture material. A comparative analysis was conducted using appropriate statistical tests to identify any significant differences in the tensile strength among the different materials.

Results: Significant variability in tensile strength was observed among the suture materials in newtons (N). PGLA exhibited the highest mean tensile strength (38.7 N), followed closely by PDO (37.1 N), whereas silk displayed the lowest tensile strength (32.8 N). Comparative analysis revealed significant differences in the tensile strength among the materials (p < 0.001).

Conclusion: This study provides valuable insights into the mechanical properties of the suture materials commonly used in oral surgery. These findings underscore the importance of considering tensile strength when selecting suture materials for specific clinical scenarios, thereby optimizing wound closure techniques and improving patient outcomes.

## Introduction

Sutures are fundamental components of wound closure in oral surgery and play a pivotal role in promoting tissue approximation and facilitating optimal wound-healing outcomes [1]. The selection of appropriate suture materials is crucial because it directly impacts the mechanical integrity of wound closure and the subsequent healing process [2]. Various factors, including tissue type, wound location, and patient characteristics, influence the choice of suture material for oral surgical procedures [3]. Understanding the mechanical properties of different suture materials is essential for clinicians to make informed decisions regarding their selection, optimal wound closure, and patient outcomes.

Polyglycolic acid (PGA), polyglactin 910 (PGLA), polylactic acid (PLA), polydioxanone (PDO), silk, and nylon are among the suture materials commonly used in oral surgery [4]. Each material possesses unique mechanical characteristics such as tensile strength, elasticity, and degradation profile, which dictate its suitability for specific clinical applications [5]. For instance, absorbable sutures such as PGA and PGLA are often preferred for mucosal closure because of their biodegradability and reduced risk of tissue reactions [6]. In contrast, nonabsorbable sutures such as silk and nylon may be indicated for skin closures or areas requiring prolonged tissue support [7].

Although the mechanical properties of suture materials are well-documented in the literature, there is a need for comprehensive evaluations specific to oral surgery applications. Previous studies have demonstrated variations in tensile strength among the different suture materials used in general surgery [8]. The present study aimed to assess the mechanical properties, particularly tensile strength, of various suture materials commonly used in oral surgery through an in vitro tensile strength study. By systematically evaluating the tensile strength of different suture materials, this study sought to provide valuable insights for clinicians in selecting the most appropriate suture material for specific clinical scenarios. Ultimately, optimizing the selection of suture materials based on their mechanical properties can contribute to enhanced wound closure techniques and improved patient outcomes in oral surgery.

## Materials and methods

A total of 192 samples of commonly used suture materials in oral surgery were selected for this study, including PGA, PGLA, PLA, PDO, silk, and nylon. These materials were chosen based on their widespread clinical use and the availability of various diameters commonly employed in oral surgery. 

In the tensile testing setup, a universal testing machine, specifically an Instron 5965 model (Instron, Norwood, MA, USA) was used to conduct tensile strength tests on the suture samples. This machine was chosen because of its versatility and accuracy in applying controlled tensile forces to the materials under test. Before commencing the actual testing procedure, meticulous attention was paid to calibrating the universal testing machine in strict accordance with the manufacturer's specifications. This calibration process ensured that the machine accurately measured the applied forces and provided reliable data during the testing process.

The testing apparatus was configured to include specialized grips designed to clamp suture samples securely during testing. These grips were selected to minimize any potential slippage or movement of the samples during tensile loading, thereby ensuring consistent and uniform stress distribution along the length of each suture sample. The design of the grips is crucial for maintaining the integrity of the samples and preventing premature failure or deformation during testing. By securely holding the suture samples in place, the grips facilitated the precise application of tensile forces, allowing for the accurate measurement of the maximum force required to break each sample. In the sample preparation phase, meticulous attention was paid to ensure consistency and accuracy in preparing the suture samples for testing. First, each type of suture material selected for the study was carefully inspected to ensure uniformity in the diameter and texture. This preliminary step aimed to minimize the variability between samples of the same material, thereby enhancing the reliability of the test results.

Using sterile surgical scissors, each suture material was cut to a standardized length of 15 centimeters (cm). This length was chosen to ensure consistency across all samples and to facilitate easy handling during testing. Careful consideration was given to minimizing any fraying or damage to the suture ends during the cutting process, as these could potentially affect the mechanical properties of the samples. Throughout the cutting process, strict adherence to sterile techniques was maintained to prevent contamination of suture samples. Sterile gloves and instruments were used to handle the suture materials, minimizing the risk of introducing foreign particles or microorganisms that could compromise the integrity of the samples. In total, 192 samples were prepared for testing, with 32 samples allocated to each type of suture material under investigation. This allocation ensured an adequate representation of each suture material in the study, allowing for meaningful comparisons of their mechanical properties. To conduct the tensile testing procedure, a systematic approach was followed to ensure accurate and consistent measurement of the tensile strength of the suture samples.

**Figure 1:**
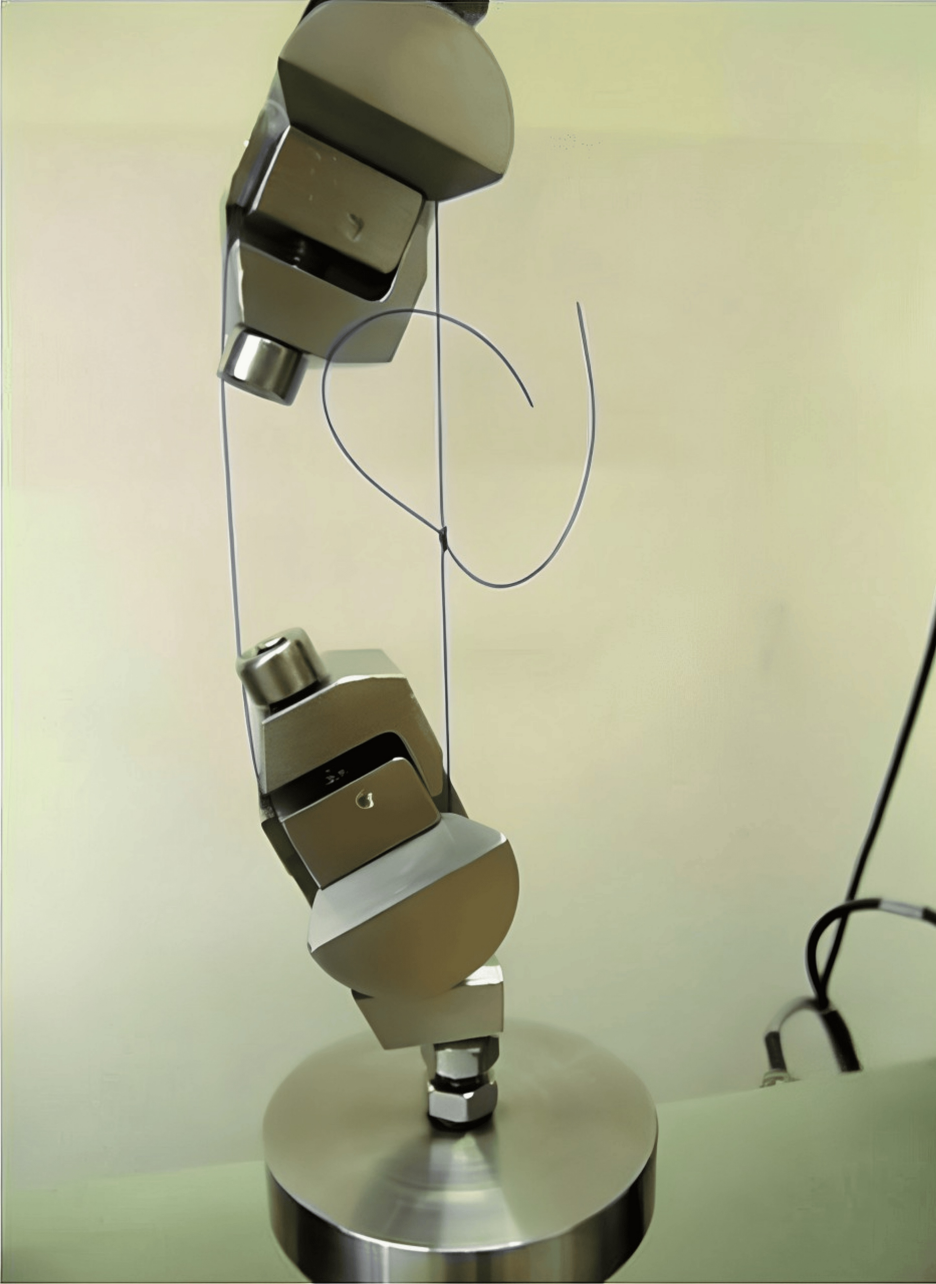
Universal testing machine for the suture thread

The universal testing machine was configured to maintain a constant crosshead speed of 50 mm/min. This speed setting was chosen based on the established protocols used in previous studies that focused on suture material testing. By maintaining a consistent crosshead speed, any variations in testing conditions that could influence the results were minimized, ensuring comparability with the existing literature. Each prepared suture sample was meticulously positioned and securely clamped within the grips of the testing machine. Special care was taken to ensure that the suture samples were uniformly aligned and centered within the grips to prevent lateral displacement during testing. Additionally, a consistent grip-to-grip distance of 5 cm was maintained for all the samples to standardize the testing conditions and facilitate the accurate measurement of tensile forces. Following proper alignment and clamping of the samples, a tensile force was applied to each sample at a constant rate by the testing machine. The force was applied steadily and uniformly to avoid sudden fluctuations that could compromise the integrity of the test results. Tension was continuously increased until failure of the suture material occurred, which was defined as the point at which the material ruptured or broke under the applied tensile load (Figure 1).

Real-time data acquisition was performed throughout the testing process to capture the load-displacement behavior of each sample. This involved monitoring and recording the changes in the force applied to the sample as well as the corresponding displacements. Real-time data acquisition allowed for the immediate observation of irregularities or anomalies during testing, enabling timely adjustments, if necessary. The universal testing machine was equipped with a software capable of automatically recording the maximum force (measured in newtons (N)) required to break each suture sample. This automated recording ensured the accuracy and precision in capturing the critical data point of the tensile strength for each sample. By documenting the maximum force at failure, comprehensive insights into the mechanical properties of the suture materials were obtained, facilitating a comparative analysis and interpretation of the results. For suture material, the maximum tensile force required to break the samples was recorded. These data were collected in real time during testing and stored electronically for subsequent analyses.

In the data analysis phase, descriptive statistics were employed to provide a comprehensive summary of the tensile strength characteristics for each suture material, encompassing measures such as mean, standard deviation, and minimum and maximum values. Additionally, a comparative analysis was conducted to discern any notable discrepancies in the tensile strength across the various suture materials. This involved employing suitable statistical tests, such as one-way analysis of variance (ANOVA) or the Kruskal-Wallis test, contingent upon the distribution of the data. Statistical significance was determined at p < 0.05, ensuring robust evaluation and interpretation of the results while discerning meaningful differences in tensile strength among the suture materials. The statistical software IBM SPSS Statistics for Windows, Version 24 (Released 2016; IBM Corp., Armonk, New York, United States) was used for data analysis.

## Results

The table presents descriptive statistics of tensile strength for the six different suture materials commonly used in oral surgery. Across all materials, the mean tensile strength ranged from 32.8 N to 38.7 N. PGLA exhibited the highest mean tensile strength of 38.7 N, followed closely by PDO with a mean tensile strength of 37.1 N. Silk displayed the lowest mean tensile strength at 32.8 N. Standard deviations ranged from 3.9 N to 5.2 N, indicating variability in tensile strength within each suture material group. The minimum and maximum tensile strength values varied between 25.3 N and 44.2 N, demonstrating a range of mechanical properties observed within each suture material (Table 1).

**Table 1:** Descriptive statistics of the tensile strength for each suture material PGA: Polyglycolic acid; PGLA: polyglactin 910; PLA: polylactic acid; PDO: polydioxanone

Suture material	Mean (N)	Standard deviation (N)	Minimum (N)	Maximum (N)
PGA	35.2	4.6	28.1	42.9
PGLA	38.7	3.9	31.5	44.2
PLA	34.5	5.2	26.9	40.1
PDO	37.1	4.2	30.5	43.6
Silk	32.8	4.8	25.3	39.4
Nylon	36.4	4.1	29.8	41.9

Both the one-way ANOVA and the Kruskal-Wallis test yielded p-values < 0.001, indicating significant differences in tensile strength among the different suture materials. This suggests that at least one pair of suture materials exhibits significantly different tensile strengths. Therefore, further post-hoc analysis is warranted to identify the specific pairs of materials that differ significantly in tensile strength (Table 2).

**Table 2:** Comparative analysis of tensile strength among suture materials ANOVA: Analysis of variance p-value less than 0.05 is significant

Statistical test	p-value
One-way ANOVA	0.0013
Kruskal-Wallis	0.0007

Post-hoc analysis using the Tukey honestly significant difference (HSD) test revealed several significant differences in tensile strength among pairs of suture materials. Notably, comparisons between PGA and PGLA, PDO, Silk, and Nylon resulted in p-values of 0.024, 0.003, <0.001, and 0.006, respectively, indicating significant differences in the tensile strength between these materials. Similarly, comparisons between PGLA and PLA, PDO, Silk, and Nylon resulted in p-values <0.05, indicating significant differences in the tensile strength between these materials. Other pairwise comparisons also yielded significant differences in tensile strength, highlighting the variability in the mechanical properties observed across different suture materials (Table 3).

**Table 3:** Post-hoc analysis of pairwise comparisons (Tukey HSD test) PGA: Polyglycolic acid; PGLA: polyglactin 910; PLA: polylactic acid; PDO: polydioxanone; HSD: honestly significant difference p-value less than 0.05 is significant

Suture materials compared	p-value
PGA vs. PGLA	0.024
PGA vs. PLA	0.116
PGA vs. PDO	0.003
PGA vs. silk	0.0007
PGA vs. nylon	0.006
PGLA vs. PLA	0.002
PGLA vs. PDO	0.04
PGLA vs. silk	0.007
PGLA vs. nylon	0.001
PLA vs. PDO	0.298
PLA vs. silk	0.029
PLA vs. nylon	0.253
PDO vs. silk	0.001
PDO vs. nylon	0.012
Silk vs. nylon	0.086

## Discussion

Suture materials play a crucial role in wound closure and healing outcomes in oral surgery. The mechanical properties of these materials, particularly their tensile strength, are fundamental considerations in selecting the most appropriate suture for specific surgical procedures. Our study investigated six commonly used suture materials: PGA, PGLA, PLA, PDO, silk, and nylon. The results revealed a significant variability in the tensile strengths of these materials. PGLA exhibited the highest mean tensile strength, followed closely by PDO, whereas silk exhibited the lowest tensile strength. These findings align with previous research indicating variations in the mechanical properties of different suture materials [9].

The variations in tensile strength observed among different suture materials have significant clinical implications. Suture materials with higher tensile strength, such as PGLA and PDO, may be preferred in oral surgical procedures where greater tissue approximation and support are required, such as in deep tissue layers or high-tension areas [10,11]. Conversely, materials with lower tensile strengths, such as silk, may be suitable for superficial closures or delicate tissues, where excessive tension could compromise tissue viability [12].

Our findings corroborate and expand upon the existing literature on the mechanical properties of suture materials. Previous studies have reported similar trends in tensile strength among various suture materials used in general surgical procedures [13,14]. However, our study focused specifically on the suture materials used in oral surgery, providing valuable insights tailored to the unique requirements of this specialty. Several factors influence the selection of suture materials in oral surgery, including the tissue type, wound location, and patient factors. For instance, absorbable sutures such as PGA and PGLA are often preferred for mucosal closures because of their biodegradability and reduced risk of tissue reactions [15]. Conversely, nonabsorbable sutures such as silk and nylon may be used in areas requiring prolonged tissue support, or where rapid absorption is not necessary, such as in skin closures [16].

The mechanical properties of suture materials directly affect wound healing outcomes. Sutures with appropriate tensile strength promote proper wound approximation and hemostasis, thereby minimizing the risk of dehiscence and infection [17]. Additionally, sutures that maintain their strength over time can provide prolonged tissue support and facilitate healing [1]. Therefore, selecting suture materials with optimal mechanical properties is essential for promoting favorable wound-healing outcomes in oral surgery.

Despite the valuable insights provided by this study, several limitations should be acknowledged. First, the in vitro nature of this study may not fully replicate the complex physiological environment of in vivo wound healing. Additionally, factors such as suture diameter, needle type, and tissue characteristics were not addressed in our study but could influence the mechanical performance of suture materials [9]. Future research should explore these factors in more detail to provide a comprehensive understanding of suture material selection in oral surgery.

## Conclusions

This in vitro study provides a detailed comparative analysis of the tensile strength of six commonly used suture materials in oral surgery: PGA, PGLA, PLA, PDO, silk, and nylon. The findings revealed significant variability in tensile strengths, with PGLA and PDO demonstrating the highest tensile strengths, making them preferable for high-tension areas and deeper tissue layers where greater support is required. Conversely, silk, which exhibited the lowest tensile strength, may be better suited for superficial closures or delicate tissues. These results highlight the importance of selecting suture materials based on their mechanical properties to optimize surgical outcomes. The study underscores that clinicians should consider specific clinical scenarios, including tissue type and wound location, to enhance wound closure techniques and improve patient outcomes in oral surgery.

## References

[1] Davis B, Smith KD (2024). Oral Surgery Suturing. https://pubmed.ncbi.nlm.nih.gov/34283455/.

[2] Faris A, Khalid L, Hashim M (2022). Characteristics of suture materials used in oral surgery: systematic review. Int Dent J.

[3] Javed F, Al-Askar M, Almas K, Romanos GE, Al-Hezaimi K (2012). Tissue reactions to various suture materials used in oral surgical interventions. ISRN Dent.

[4] O'Neal RB, Alleyn CD (1997). Suture materials and techniques. Curr Opin Periodontol.

[5] Tajirian AL, Goldberg DJ (2010). A review of sutures and other skin closure materials. J Cosmet Laser Ther.

[6] Pillai CK, Sharma CP (2010). Review paper: absorbable polymeric surgical sutures: chemistry, production, properties, biodegradability, and performance. J Biomater Appl.

[7] Rose J, Tuma F (2024). Sutures and Needles. https://www.ncbi.nlm.nih.gov/books/NBK539891/.

[8] Polykandriotis E, Daenicke J, Bolat A, Grüner J, Schubert DW, Horch RE (2022). Individualized wound closure-mechanical properties of suture materials. J Pers Med.

[9] Alizadeh-Osgouei M, Li Y, Wen C (2019). A comprehensive review of biodegradable synthetic polymer-ceramic composites and their manufacture for biomedical applications. Bioact Mater.

[10] Byrne M, Aly A (2019). The surgical suture. Aesthet Surg J.

[11] Chu CC (1981). Mechanical properties of suture materials: an important characterization. Ann Surg.

[12] Greenberg JA, Clark RM (2009). Advances in suture material for obstetric and gynecologic surgery. Rev Obstet Gynecol.

[13] Taysi AE, Ercal P, Sismanoglu S (2021). Comparison between tensile characteristics of various suture materials with two suture techniques: an in vitro study. Clin Oral Investig.

[14] Kuzu TE (2022). Comparison tensile strength of different suture materials. Cumhuriyet Dent J.

[15] Xu L, Liu Y, Zhou W, Yu D (2022). Electrospun medical sutures for wound healing: a review. Polymers (Basel).

[16] Islam A, Ehsan A (2011). Comparison of suture material and technique of closure of subcutaneous fat and skin in caesarean section. N Am J Med Sci.

[17] Azmat CE, Council M (2024). Wound Closure Techniques. https://www.ncbi.nlm.nih.gov/books/NBK470598/.

